# International Collaboration is the Only Way to Protect Ourselves from the Next Pandemic

**DOI:** 10.1007/s10393-022-01609-4

**Published:** 2022-08-08

**Authors:** Peter Daszak

**Affiliations:** EcoHealth Alliance, 520 Eighth Avenue, NY, 10018 USA

The COVID-19 pandemic has had a profound impact on global health and well-being during the last 2 and a half years. It has also laid bare just how devastating pandemics can be—shaking national economies, revealing deep-seated inequities among and within nations, fracturing global health security and threatening international collaboration among health scientists.

As we climb our way carefully out of COVID-19, we’re experiencing the reality that new diseases, and the pandemics they cause, are on the rise. We’re seeing old foes like avian flu (Anthens 2022) spread into new species. We’re seeing monkeypox shift from a growing concern in endemic countries to become a global threat that triggered the WHO committee on public health emergencies of international concern (PHEIC) to meet for the first time since it declared COVID-19 a pandemic in early 2020, only to declare that monkeypox should now also be considered a PHEIC. After 2 years of careful social distancing, formerly common ailments are causing more serious disease (Sellers 2022) in children whose immune systems have been sheltered from their normal exposure.

Analysis of pandemic beginnings, including work at EcoHealth Alliance (Jones et al. 2008), shows that the microbes that cause them have been spilling over from animals to people at an exponentially increasing rate for the last few decades. They’re rising in tandem with our own impacts on the planet (Allen et al. 2017)—land use change, deforestation, agricultural expansion, climate change (Carlson et al. 2022), urbanization, and the global wildlife trade.

Behind every global pandemic is a humble origin—often a single person infected by an animal virus—an event that brings an unfortunate mix of the right type of virus affecting the right person at the right time to spread across communities, landscapes and countries. Identifying exactly where and when this critical spillover happens is surely the key to preventing future pandemics. We already know roughly where these spillovers tend to happen—in emerging disease hotspots where people and diverse wildlife live in rapidly changing environments. We have a good idea of how many viruses have the potential to emerge (a few hundred thousand or so) (Carroll et al. 2018) and which animal hosts carry the most (Olival et al. 2017) (bats, rodents, primates). But up until now, the scale of these spillovers has been anybody’s guess.

In a paper (Sanchez et al. 2022) just published in Nature Communications, we show for the first time how hard defeating pandemics will be. We analyzed all known reports of viruses related to SARS and SARS-CoV-2 and mapped out the distribution of each species of bats in which they have been reported. This covers a region of South China, Southeast and South Asia that’s home to over 499 million people. We then canvassed the scientific literature to identify every study that examined people in this region for antibodies to bat viruses—evidence that in their lifetimes they’ve been infected by a new bat virus. We used these, and data on how long antibodies last, to estimate that over 66,000 people are likely infected each year with bat-origin coronaviruses of the type that caused SARS and COVID-19. With only a handful of outbreaks known, it looks like 99.9% of spillovers are dead ends—caused by viruses not able to make people sick, or causing illness or even outbreaks that get passed over or never reported.

How can we find the one-in-a-few-hundred-thousand spillover events that could actually lead to the next pandemic? This will require a new type of ‘smart surveillance’ that is targeted, collaborative, and science-based. First, **we will need to triage our efforts to maximize our chances of success**. This means working in disease hotspots where wildlife species carry the highest number of viruses that are likely able to infect people; where communities are at highest risk of picking up the next spillover; and where there is the least capacity to identify potentially harmful viruses. This means working with countries to help build healthcare capacity in remote areas so that every case of a new disease is investigated. It means working with communities—often poor and marginalized—who rely on their contact with wildlife and livestock for their survival.

Second, **we will need to bring the world’s best scientists together to collaborate in a more systematic fashion**. This will involve scaling up new serological tools (Kupferschmidt 2020) that can both distinguish between known and novel viral agents and tease apart those who’ve been vaccinated against COVID-19 in Southeast Asia from those who’ve picked up a new bat-origin virus. It will mean better coordination among clinics so that samples from people with undiagnosed illnesses can be identified rapidly using state-of-the-art viral sequencing. All these efforts will provide critical raw data for the many new pandemic forecasting initiatives (Sun 2022) now launched as a response to COVID-19 around the world. Cooperating with countries in disease hotspots will provide the fastest and most reliable data to fuel the AI models that can alert us all when a disease begins to spread.

Third, **we need to understand why diseases are spilling over and try to reduce the causes**. Surveys conducted by social scientists help identify the risk behaviors that drive exposure and also help us understand the incentives to reduce these behaviors, so that effective intervention strategies can be developed. Larger scale drivers like land use change (Daszak 2020) and unregulated wildlife trade (Gorman 2020) are beginning to be tackled by a combination of localized efforts to promote sustainable harvests, and national and international policies to curtail excesses. Companies that are part of the value chain for timber, agricultural products like palm oil, and wildlife products (like the fashion industry) will all need to play their part—as will we, the consumers of these products.

And finally, **all of this will take something that’s under the greatest threat today—international cooperation**. A key lesson we should have learned as the whole planet locked down in March 2020, is that a virus that emerges ‘over there’ affects us all. By failing to block the early chains of spread, we gave ourselves the far harder task of controlling coronavirus infection on a global scale. The irony of the COVID-19 pandemic is that, by emerging at a time of fractured geopolitics, it neatly undermined our ability to deal with it. COVID-19 built on our fears and prejudice so that every aspect of the response, from analyzing its origins, complying with public health measures to counter transmission, through to the uptake of vaccinations and treatments, became part of a polarizing political debate. But, as we’ve learned the hard way, viruses respect neither geographic boundaries nor political parties—all that matters to viruses is that they find new humans to infect. To defeat the next pandemic and build a resilient future that can look backwards on this ‘age of pandemics’ will require a similarly single-minded focus on following the science and collaborating with our friends and competitors alike.
